# High prevalence of anxiety, depression, and stress among remote learning students during the COVID-19 pandemic: Evidence from a meta-analysis

**DOI:** 10.3389/fpsyg.2022.1103925

**Published:** 2023-01-10

**Authors:** Tianyuan Xu, Huang Wang

**Affiliations:** ^1^School of Foreign Languages, Zhejiang Gongshang University, Hangzhou, China; ^2^School of Foreign Studies, Hunan First Normal University, Changsha, China

**Keywords:** COVID-19, prevalence, mental health, anxiety, depression, stress, remote learning students, meta-analysis

## Abstract

The coronavirus disease 2019 (COVID-19) pandemic has influenced all aspects significantly, and an estimated 1.5 billion students across the globe have been forced to keep up with online courses at home. Many recent empirical studies reported the prevalence of mental health problems among students caused by remote learning during the COVID-19 pandemic, but a few studies aggregated these results. Therefore, to strengthen statistical power, the article aimed to examine the prevalence of anxiety, depression, and stress among remote learning students during the COVID-19 pandemic *via* a meta-analysis. A total of 36 original articles have been selected from five academic databases, including Web of Science, PubMed, Scopus, EBSCO, and Google Scholar, covering 78,674 participants in 19 nations, and yielding 60 effect sizes (22 for anxiety, 17 for depression, and 21 for stress) based on the random effects model *via* Comprehensive Meta-Analysis (CMA) software. The results showed that the prevalence of anxiety, depression, and stress among remote learning students during the COVID-19 pandemic was as high as 58, 50, and 71%, respectively. Besides, the moderator analysis found that (1) the prevalence of anxiety and depression among students in higher education was significantly higher than that of students in elementary education. (2) an increasing number of medical students and students in emergency remote learning context suffered from mental stress than their non-medical and traditional distance learning counterparts. Thus, the COVID-19 pandemic triggers concerns related to physical health and mental disorders, especially for remote online learning students. The current situation should be brought to the forefront by educators to develop psychological interventions for relieving students’ anxiety, depression, and stress during the pandemic period.

## 1. Introduction

In December 2019, a cluster of pneumonia cases caused by a newly discovered coronavirus was reported in Wuhan, China. The disease was named coronavirus disease 2019 (COVID-19), which first spread across China and then across 216 nations, areas, or territories worldwide (Dutta et al., 2020). Facing the rapid spread of the disease, the World Health Organization (WHO) declared the COVID-19 outbreak a Public Health Emergency of International Concern on 30 January 2020 and a pandemic on 12 March 2020 (WHO, 2020). According to the statistics reported by United Nations Educational, Scientific and Cultural Organization (UNESCO), schools and universities have been closed in around 190 countries across the world to prevent and control the spread of the COVID-19 pandemic, which leads to an estimated 1.5 billion students of all ages having been forced to stay at home and keep up with lessons online (WHO, 2022).

Online education, including online teaching and learning, refers to a new style that involves no in-person communication but digital online interaction between students and instructors (Tunc and Toprak, 2022). Effective online education requires careful planning and preparation not only for course content but also for providing support for different types of digital interactions (Fuchs, 2022). However, the outbreak of the COVID-19 pandemic broke the traditional model of online learning. A new concept, emergency remote learning was put forward and refers to a temporary switch of instruction delivery *via* an alternate delivery mode because of crisis circumstances (Tunc and Toprak, 2022). One of the major primary misalignments between online education and emergency remote education is that the latter is usually improvised with little time to plan and prepare due to time constraints (Ferri et al., 2020; Fuchs, 2022).

Although online education possesses distinct advantages, such as saving commuting time and flexibility to choose, disadvantages such as online study potentially influencing academic performance and the lack of practical knowledge have also been found (Ferri et al., 2020; Kotrikadze and Zharkova, 2021). Apart from the negative effect exerted on the study itself, distance learning also causes many mental problems, such as online anxiety (anxiety aroused from acquiring knowledge through the use of the internet; Warner et al., 2020) and technostress (stress aroused from the prolonged exposure of information-communication-technologies; Torales et al., 2022). After the outbreak of the COVID-19 pandemic, many scholars across the globe have paid closer attention to the mental health of students who have undergone (emergency) online learning. For example, in China, the prevalence of symptoms of anxiety, depression, and stress was observed in 32.9, 31.9, and 14.6%, respectively, of university medical students (Chang et al., 2021), and 25.13, 29.95, and 75.89% of university students from different majors suffered from anxiety, depression, and insomnia symptoms, respectively (Zhang et al., 2022). In Poland, a total of 56 and 58% of the students from the Opole University of Technology were characterized by depression and stress symptoms, and even 18% of the participants had suicidal thoughts (Rutkowska et al., 2022). Despite numerous empirical studies focusing on the detection rate of mental problems among remote learning students during the COVID-19 pandemic, a few studies integrated the results of these multiple studies that tend to address the same question. Therefore, to aggregate data resulting in a more substantial statistical power than any individual study, the study aimed to examine the prevalence of anxiety, depression, and stress among remote learning students during the COVID-19 pandemic *via* a meta-analysis.

## 2. Methods

This study aimed to explore the prevalence of anxiety, depression, and stress among remote learning students during the COVID-19 pandemic. To achieve this aim, previous studies were searched first and screened based on the inclusion and exclusion criteria. Then, data were extracted and coded from selected empirical studies. Statistical analyses including testing and correcting publication bias, selecting a model, and processing data were carried out as the last procedure. The methods of the study are introduced in subsections.

### 2.1. Literature searching and screening

The present meta-analysis concentrated on the prevalence of anxiety, depression, and stress among remote learning students during the COVID-19 pandemic. The first step in conducting this study is searching and screening the previous studies on this well-specific subject. Academic databases, including Web of Science, PubMed, Scopus, EBSCO, and Google Scholar, were used to search related literature systematically. To identify the articles, search terms (i.e., COVID-19, Coronavirus, Online learning, emergency online learning, remote learning, distance learning, e-learning, mental health, anxiety, depression, and stress) and all the possible combinations of these keywords were input in the search bar with the following string: (“COVID-19” OR “Coronavirus”) AND (“Online learning” OR “emergency online learning” OR “remote learning” OR “distance learning” OR “e-learning”) AND (“mental health” OR “anxiety” OR “depression” OR “stress”). The final search date of this study was 1 November 2022.

**Figure 1:**
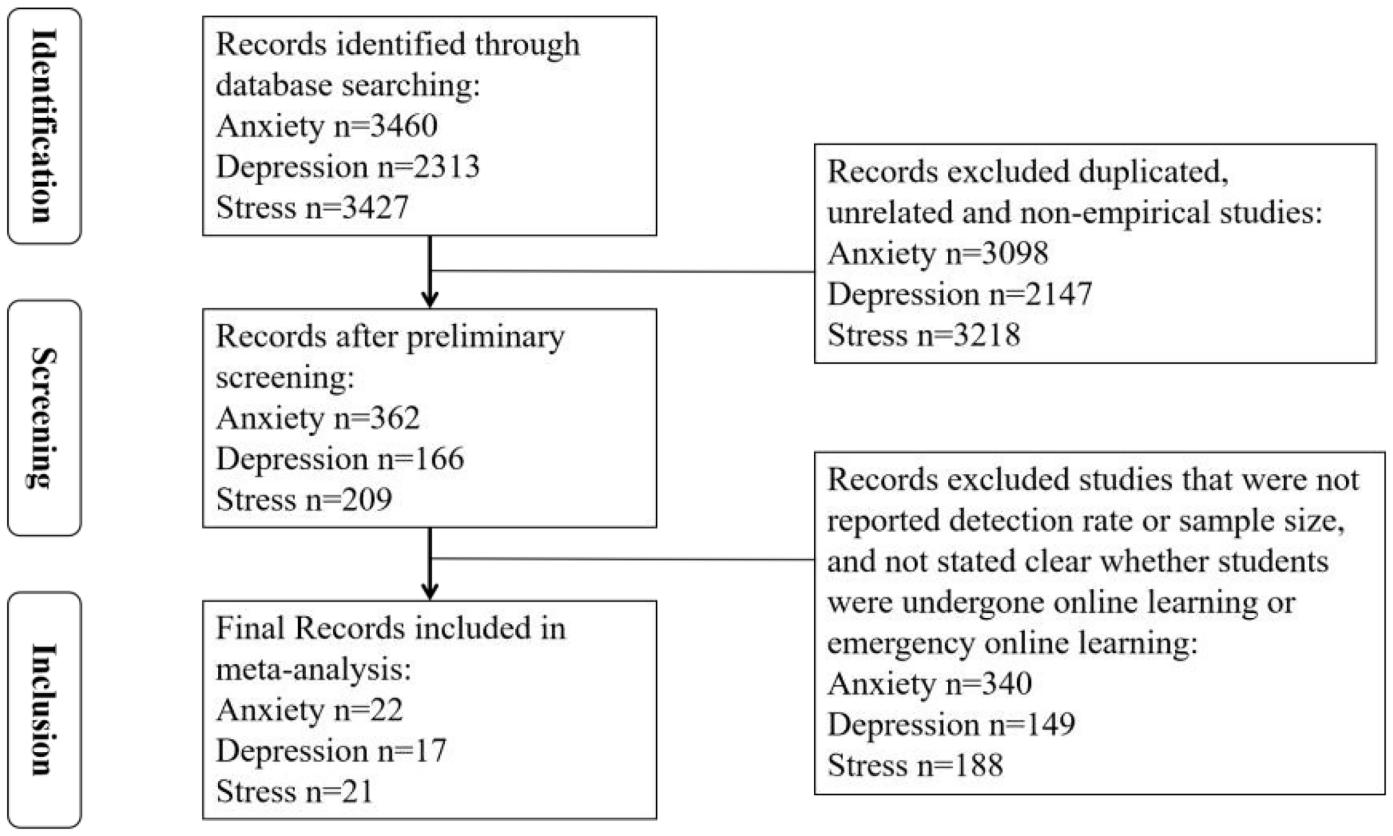
A flow diagram of literature searching and screening.

The criteria for screening resulting articles were as follows: (1) studies that were published during the COVID-19 pandemic; (2) studies that were empirical research; (3) studies that reported the detection rate of anxiety, depression, or stress among remote learning students during the COVID-19 pandemic; (4) studies that reported the accurate sample size; and (5) studies that specified whether students had undergone online learning or emergency online learning. After applying these inclusion criteria, a total of 36 studies, yielding 22 anxiety effect sizes, 17 depression effect sizes, and 21 stress effect sizes, were selected in this meta-analysis (see flow diagram of Figure 1).

### 2.2. Data extraction and coding

Selected studies were coded, and data were extracted according to the following variables: author, publication year, country, learning phrase, students’ major, whether attending emergency online learning, measurement, sample size, and prevalence rate. Tables 1–3 show the summary coding of included studies on anxiety, depression, and stress.

**Table 1:** Summary coding of included anxiety studies (arranged in the alphabetical order of the first author).

First author	Year	Country	Learning phrase	Students’ major	Whether attending emergency online learning	Measurement	Sample size	Rate
Acosta	2021	USA	K-12	/	Yes	Self-report	145	51.80%
Biber	2019	USA	University	/	No	GAD-7	1,640	92.62%
Bolatov	2021	Kazakhstan	University	Medical	No	GAD-7	798	42.60%
Chang (a)	2021	China	University	Medical	No	DASS-21	4,115	32.90%
Chang (b)	2021	China	University	Diverse	No	DASS-21	5,558	36.32%
Demaray	2022	USA	K-12	/	No	GAD-7	2,738	41.16%
Dirzyte	2021	Lithuania	University	/	No	GAD-7	444	62.30%
Elshami	2021	UAE	University	Medical	No	Online Course Anxiety Scale	358	37.70%
Fawaz	2020	Lebanon	University	/	No	DASS-21	520	43.80%
Halat	2022	Lebanon	University	Medical	No	DASS-21	561	70.70%
Hoque	2021	Bangladesh	University	/	No	Zung’s Scale	206	82.50%
Hu	2022	China	University	/	No	GAD-2	512	72.00%
Li	2022	China	University	Diverse	No	Self-designed	622	66.00%
Liu	2022	China	University	/	No	DASS-21	1,506	38.00%
Moy	2021	Malaysia	University	/	No	DASS-21	310	51.28%
Peng	2022	China	K-12	/	No	GAD-7	39,751	10.30%
Perkins	2021	USA	K-12	/	No	GAD-2	320	22.80%
Rodrigues	2022	Portugal	University	Medical	No	/	415	84.50%
Srivastava	2021	India	University	Medical	Yes	GAD-7	97	56.70%
Toprak	2022	Turkey	University	Medical	Yes	PSS-10	2,290	100%
Torales	2022	Paraguay	University	Diverse	No	GAD-7	378	87.10%
Zhang	2022	China	University	/	No	GAD-7	788	25.13%

/ means the information was unreported; GAD-7, Generalized Anxiety Disorder 7-item; GAD-2, Generalized Anxiety Disorder 2-item; PSS-10, Perceived Stress Scale 10-item; and DASS-21, The Depression, Anxiety and Stress Scale 21-item.

**Table 2:** Summary coding of included depression studies (arranged in alphabetical order of the first author).

Author	Year	Country	Learning phrase	Students’ major	Whether attending emergency online learning	Measurement	Sample size	Rate
Acosta	2021	USA	K-12	/	Yes	Self-report	145	44.80%
Azmi	2022	SA	University	Diverse	No	Zung’s Scale	157	75.00%
Bolatov	2021	Kazakhstan	University	Medical	No	PHQ-9	798	80.30%
Chang (a)	2021	China	University	Medical	No	DASS-21	4,115	31.90%
Chang (b)	2021	China	University	Diverse	No	DASS-21	5,558	35.15%
Demaray	2022	USA	K-12	/	No	CES-DC	2,738	41.16%
Dirzyte	2021	Lithuania	University	/	No	PHQ-9	444	75.70%
Fawaz	2020	Lebanon	University	/	No	DASS-21	520	33.40%
Halat	2022	Lebanon	University	Medical	No	DASS-21	561	64.00%
Islam	2020	Bangladesh	University	/	No	PHQ-9	476	82.30%
Liu	2022	China	University	/	No	DASS-21	1,506	36.06%
Moy	2021	Malaysia	University	/	No	DASS-21	310	29.40%
Perkins	2021	USA	K-12	/	No	PHQ-2	320	19.40%
Rutkowska (a)	2021	Slovakia	University	/	No	BDI	3,051	47.00%
Rutkowska (b)	2022	Poland	University	Diverse	No	BDI	753	56.00%
Torales	2022	Paraguay	University	Diverse	No	PHQ-2	378	60.30%
Zhang	2022	China	University	/	No	PHQ-9	788	29.95%

/ means the information was unreported; PHQ-9, Patient Health Questionnaire 9-item; PHQ-2, Patient Health Questionnaire 2-item; DASS-21, The Depression, Anxiety and Stress Scale 21-item; CES-DC, Epidemiological Studies Depression Scale for Children; and BDI, Beck Depression Inventory.

**Table 3:** Summary coding of included stress studies (arranged in alphabetical order of the first author).

Author	Year	Country	Learning phrase	Students’ major	Whether attending emergency online learning	Measurement	Sample size	Rate
Amerson	2021	USA	University	Medical	No	PSS-10	256	98.00%
Azmi	2022	SA	University	Diverse	No	Zung’s Scale	157	75.00%
Chang (a)	2021	China	University	Medical	No	DASS-21	4,115	14.60%
Chang (b)	2021	China	University	Diverse	No	DASS-21	5,558	17.24%
Fawaz	2020	Lebanon	University	/	No	DASS-21	520	12.70%
Halat	2022	Lebanon	University	Medical	No	DASS-21	561	48.30%
Hegler	2022	USA	University	Diverse	Yes	Self-designed	538	89.00%
Kabir	2021	Bangladesh	University	/	No	PSS	1,145	90.92%
Liu	2022	China	University	/	No	DASS-21	1,506	24.44%
Motappa	2022	India	University	Medical	No	PSS	324	96.00%
Moy	2021	Malaysia	University	/	No	DASS-21	310	56.50%
Nikas	2021	Cyprus	University	Medical	Yes	PSS-10	173	77.30%
Pieh	2021	Austria	K-12	/	No	PSS-10	2,884	89.00%
Quintiliani	2021	Italy	University	Medical	No	PSS-10	955	89.40%
Radwan	2021	Palestine	K-12	/	No	PSS	385	71.20%
Rutkowska (a)	2021	Slovakia	University	/	No	PSS-10	3,051	98.00%
Rutkowska (b)	2022	Poland	University	Diverse	No	PSQ	753	58.17%
She	2021	China	K-12	/	No	Self-designed	3,136	6.40%
Toprak	2022	Turkey	University	Medical	Yes	PSS-10	2,290	94.80%
Torales	2022	Paraguay	University	Diverse	No	TechQ	378	52.60%
Wang	2021	China	University	Medical	No	PSS-10	369	82.30%

/ means the information was unreported; DASS-21, The Depression, Anxiety and Stress Scale 21-item; TechQ, Technostress Questionnaire; PSS, Perceived Stress Scale; and PSS-10, Perceived Stress Scale 10-item.

### 2.3. Statistical analyses

Testing publication bias, selecting a model, and processing data are indispensable to statistical analysis before conducting meta-analysis. In terms of publication bias, it indicates that studies with more significant findings are more likely to be published in a scientific journal, so it is a severe issue that can undermine the reliability and validity of results in a meta-analysis (Begg and Berlin, 1988; Lin and Chu, 2017). One method to test publication bias is observing the asymmetry of the funnel plot. However, due to the lack of precision, multiple statistical tests have been put forward to examine publication bias accurately, such as Egger’s regression test (Egger et al., 1997) and Begg’s rank test (Begg and Mazumdar, 1994). If publication bias is detected, methods can be applied to correct bias, including the trim-and-fill method (Duval and Tweedie, 2000) or Rosenthal’s failsafe N (Rosenthal, 1979). In this study, Egger’s regression test was chosen to be the method applied to assess whether publication bias exists in this meta-analysis.

As regards model selection, two statistical models have been modified in the meta-analysis: the fixed effects model and the random effects model (Hedges and Vevea, 1998). The former fixed effects model treats the effect size parameters as fixed but unknown constants to be estimated, while the latter random effects model treats the effect size parameters as though they were a random sample from the population of effect parameters (Rosenthal and Rubin, 1982; DerSimonian and Laird, 1986). To decide which model (fixed effects model vs. random effects model) should be applied, measuring heterogeneity through the Q test and I^2^ confidence interval (CI) are crucial evaluation criteria. If the Q test shows significant or I^2^ results higher than 75%, it indicates that high heterogeneity exists, and a random effects model should be selected; otherwise, a fixed effects model should be chosen (Huedo-Medina et al., 2006).

This meta-analysis was processed and performed using the Comprehensive Meta-Analysis (CMA) software. To aggregate the overall prevalence rate, the software first transformed input ratio data into logit data through the formula logit=log(p/(1−p)) , and then, converted logit data back to ratio data by the formula $\mathrm{var}\left(\mathrm{logit}\right)=\frac{1}{\mathrm{case}}+\frac{1}{\mathrm{non}-\mathrm{case}}$ (Card, 2012). There were two ways to analyze moderating effect in this meta-analysis: (1) When moderating variables were continuous, meta-regression was used to test whether the results were significant and (2) when moderating variables were categorical; the subgroup analysis was used to test whether the results were significant. In the subgroup analysis, to ensure the representativeness of the studies under certain moderating variables, the number of effect sizes under the same moderating variable should be no less than 3 (Zhang et al., 2021).

## 3. Results

The results of the study are presented in the following subsections. The Results section starts with description of the characteristic of the study sample, followed by an investigation and measurement of heterogeneity and publication bias. The Results section ends in displaying the combined effect, sensitivity, and moderator analysis of the present meta-analysis.

### 3.1. Study sample characteristics

Overall, 60 total effect sizes from 36 different studies were extracted, and 78,674 participants were included in this meta-analysis. Specifically, anxiety included 22 studies and 64,072 participants, generating 22 effect sizes; depression comprised 17 studies and 22,618 participants, yielding 17 effect sizes; stress incorporated 21 studies and 29,364 participants, generating 21 effect sizes. These 36 studies were conducted from 19 nations (i.e., Austria, Bangladesh, China, Cyprus, India, Italy, Kazakhstan, Lebanon, Lithuania, Malaysia, Palestine, Paraguay, Poland, Portugal, Saudi Arabia, Slovakia, Turkey, United Arab Emirates, and the United States of America), covering both developed and developing countries from Asia, Europe, North America, and South America.

### 3.2. Investigating heterogeneity and publication bias

Investigating heterogeneity is the critical step to judge whether the fixed or random effects model should be selected in this meta-analysis. Table 4 shows that all Q tests were significant, and I^2^ results exceeded 75% in anxiety (*p* = 0.000, *I^2^* = 99.784), depression (*p* = 0.000, *I^2^* = 98.955), and stress (*p* = 0.000, *I^2^* = 99.797) studies, indicating that the random effects model should be chosen as the statistical model in this study (Huedo-Medina et al., 2006).

**Table 4:** Heterogeneity and publication bias test.

Mental health	Heterogeneity		Egger’s
	*p*	*I^2^*	*p*
Anxiety	0.00	99.78	< 0.001
Depression	0.00	98.96	0.04
Stress	0.00	99.80	0.01

**Figure 2:**
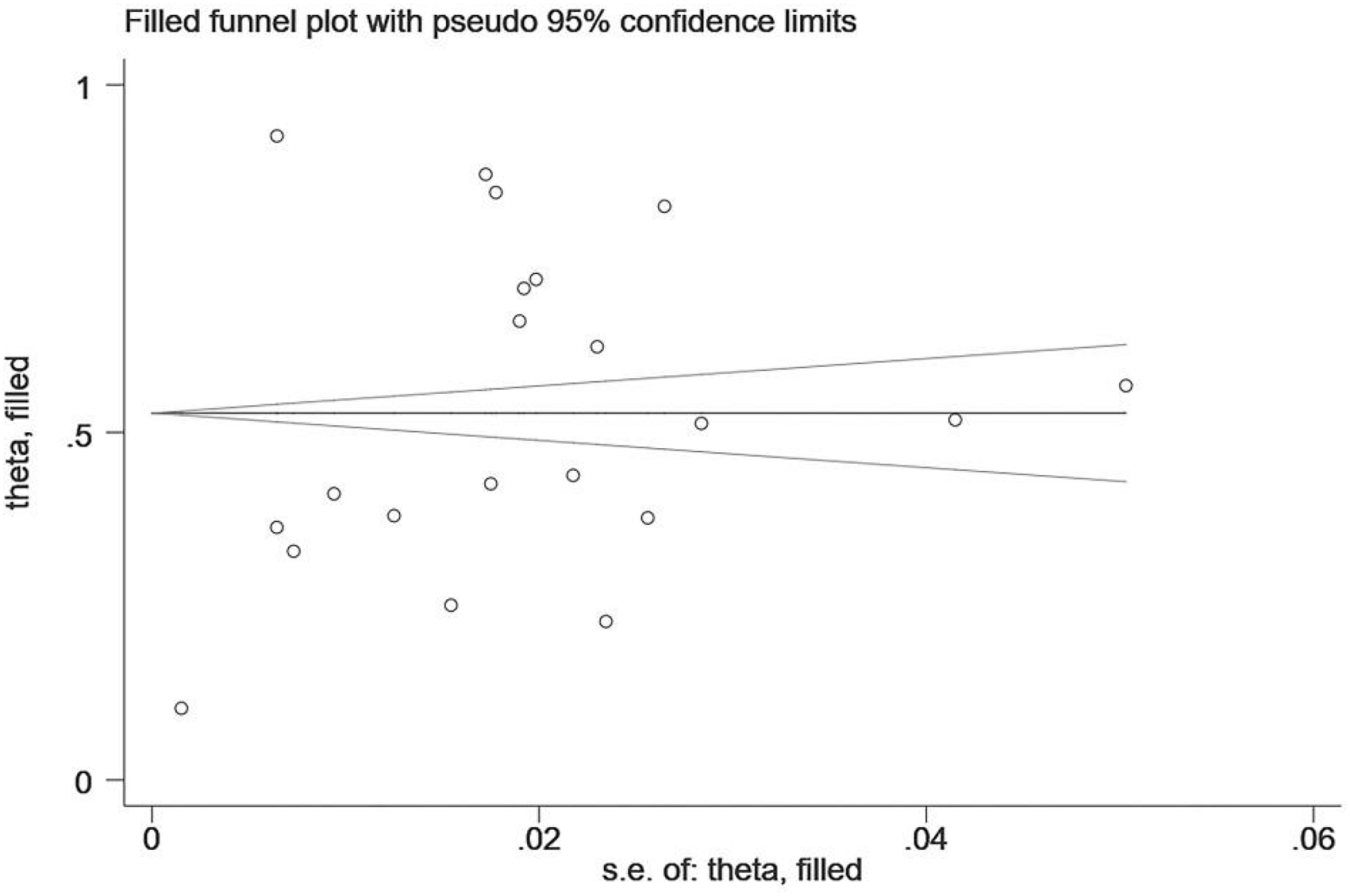
A funnel plot assessing publication bias of the prevalence of anxiety among remote learning students during the COVID-19 pandemic.

**Figure 3:**
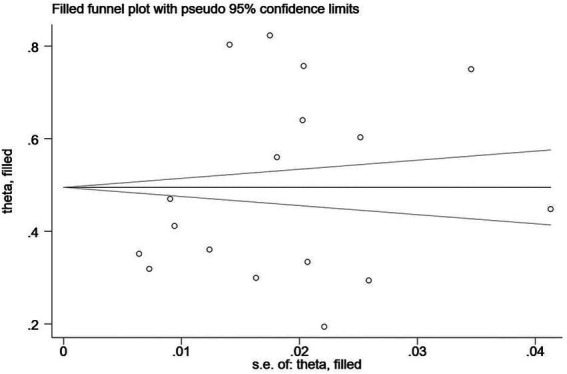
A funnel plot assessing publication bias of the prevalence of depression among remote learning students during the COVID-19 pandemic.

**Figure 4:**
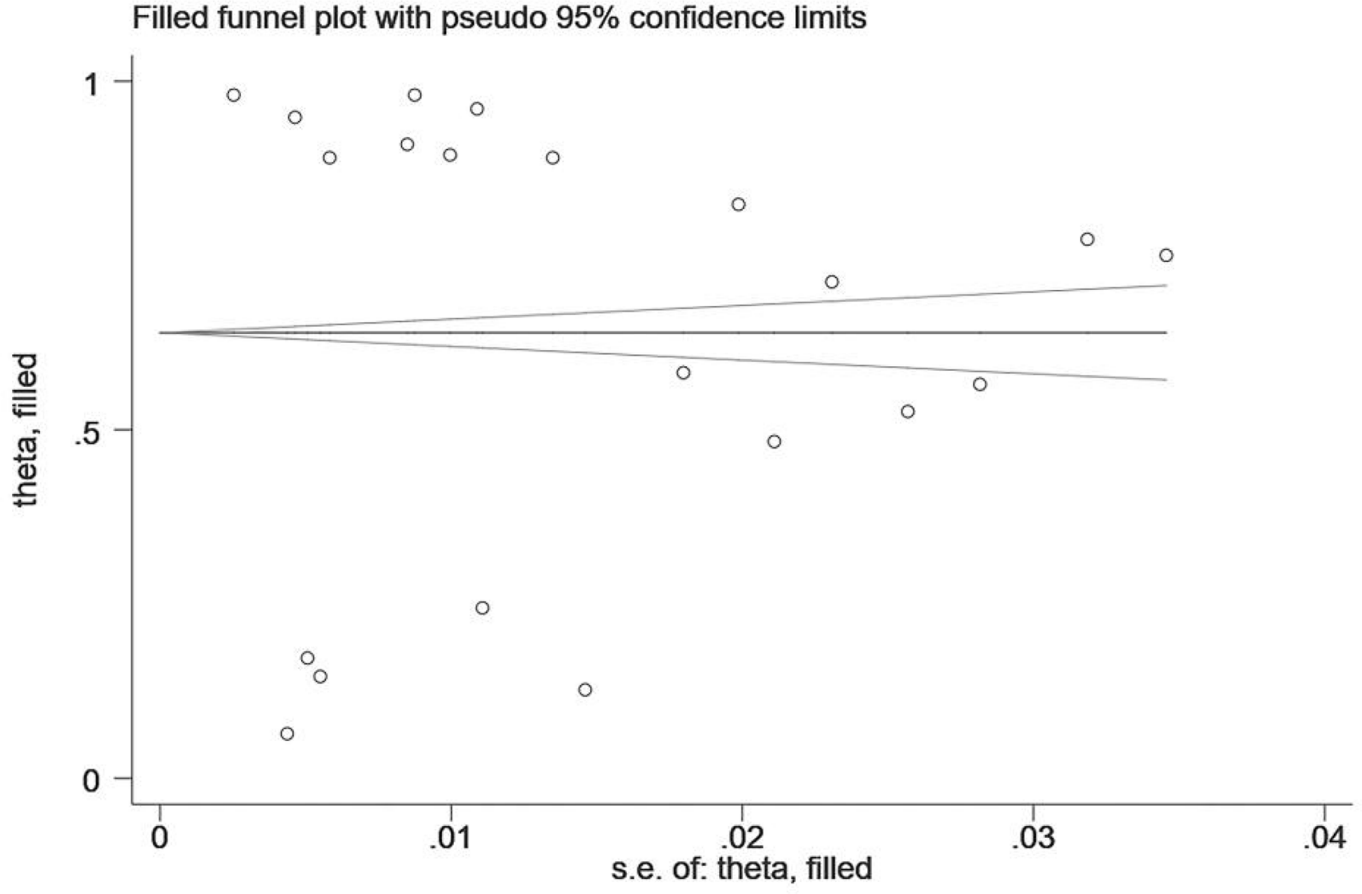
A funnel plot assessing publication bias of the prevalence of stress among remote learning students during the COVID-19 pandemic.

In addition, Egger’s regression test is indispensable for examining the publication bias in this study. As shown in Table 4, the value of *p* showed significance in anxiety (*p* < 0.001), depression (*p* = 0.04), and stress (*p* = 0.04), with publication bias manifesting in any of the three clinical symptoms. Therefore, the trim-and-fill method, one of the most popular means, was opted for correcting publication bias (Shi and Lin, 2019). Trim-and-fill analysis was completed in the software Stata. The analysis showed that “no trimming performed; data unchanged,” indicating that the results were robust and that no literature were needed to be added to the present study. The funnel plots assessing publication bias of anxiety, depression, and stress are shown in Figures 2–4 in sequence.

### 3.3. Combined effect, sensitivity, and moderator analysis

Based on the random effects model, the prevalence of the mental problem is shown in Table 5: (1) the prevalence of anxiety among remote learning students during the COVID-19 pandemic was 58% [95% confidence interval (CI) = [0.45, 0.70]]; (2) the prevalence of depression among remote learning students during the COVID-19 pandemic was 50% (95% CI = [0.43, 0.57]); and (3) the prevalence of stress among remote learning students during the COVID-19 pandemic was 71% (95% CI = [0.52, 0.84]).

**Table 5:** The prevalence of anxiety, depression, and stress among remote learning students during the COVID-19 pandemic.

Mental health	*k*	Sample size	Effect size and 95% CI		
			*r*	Lower limit	Upper limit
Anxiety	22	64,072	0.58	0.45	0.70
Depression	17	22,618	0.50	0.43	0.57
Stress	21	29,364	0.71	0.52	0.84

Sensitivity analysis was also applied to examine the robustness of the results. After a random individual study was removed from the input data, the prevalence of anxiety fluctuated between 54.0 and 60.0%, depression between 47.4 and 51.9%, and stress between 69.5 and 73.3%. There is little difference between the finding before and after sensitivity analysis, which indicates that the results are robust and stable.

In terms of moderator analysis, several variables were set as moderator types to assess whether a third variable causes a significant difference. In terms of anxiety (see Table 6), the results showed that (1) the moderating effect of publication year was nonsignificant (*b* = −0.13, 95% CI = [−0.75, 0.50]), which indicates that the prevalence of anxiety hardly changed with time lapse; (2) the moderating effect of whether attending emergency online learning (*p* = 0.09), development level (*p* = 0.63), and students’ major (*p* = 0.92) were all not significant; and (3) the moderating effect of educational level (*p* = 0.03) was significant. Prevalence of the anxiety of university students (63%) was more than two times that of the k-12 students (29%).

**Table 6:** Moderator analysis for the prevalence of anxiety.

Moderator variable	Heterogeneity			Type	*k*	*r*	95% CI	
	*Q*	*df*	*p*				Lower limit	Upper limit
Whether attending emergency online learning	2.97	1	0.09	Yes	3	0.81	0.52	0.94
				No	19	0.54	0.40	0.67
Educational level	4.89	1	0.03	Elementary	4	0.29	0.11	0.58
				Tertiary	18	0.63	0.53	0.72
Development level	0.24	1	0.63	Developed	6	0.63	0.37	0.84
				Developing	16	0.58	0.41	0.69
Student major	0.01	1	0.92	Diverse	3	0.66	0.35	0.88
				Medical	7	0.64	0.47	0.78

As regards depression (see Table 7), the results showed that (1) the moderating effect of publication year was non-significant (*b* = −0.13, 95% CI = [−0.75, 0.49]), which also indicates that the prevalence of depression hardly changed over time; (2) the moderating effect of development level (*p* = 0.40) and students’ major (*p* = 0.92) were both not significant; and (3) the moderating effect of educational level (*p* = 0.04) was significant. The prevalence of depression in university students (53%) was much higher than that of k-12 students (34%).

**Table 7:** Moderator analysis for the prevalence of depression.

Moderator variable	Heterogeneity			Type	*k*	*r*	95% CI	
	*Q*	*df*	*p*				Lower limit	Upper limit
Educational level	4.25	1	0.04	Elementary	3	0.34	0.21	0.50
				Tertiary	14	0.53	0.45	0.62
Development level	0.72	1	0.40	Developed	5	0.45	0.35	0.57
				Developing	12	0.52	0.42	0.61
Student major	0.01	1	0.92	Diverse	3	0.66	0.35	0.88
				Medical	7	0.64	0.47	0.78

The number of studies under the emergency online learning context is less than 3, so “whether attending emergency online learning” cannot be regarded as a moderator variable in depression analysis.

For the third mental health problem, stress (see Table 8), the results showed that (1) the moderating effect of publication year was not significant (*b* = 0.63, 95% CI = [−0.80, 2.06]), which also indicates that the prevalence of stress scarcely variates with time; (2) the moderating effect of educational level (*p* = 0.59) and students’ major (*p* = 0.25) was both not significant; and (3) the moderating effect of whether attending emergency online learning (*p* = 0.03) and development level (*p* = 0.00) was significant. The stress prevalence of students under emergency online learning contexts (89%) was much higher than that of students under traditional online learning contexts (67%). In addition, the stress prevalence of students in a developed country (93%) was more than 1.5 times higher than their counterparts in a developing country (56%).

**Table 8:** Moderator analysis for the prevalence of stress.

Moderator variable	Heterogeneity			Type	*k*	*r*	95% CI	
	*Q*	*df*	*p*				Lower limit	Upper limit
Whether attending emergency online learning	4.92	1	0.03	Yes	3	0.89	0.76	0.95
				No	18	0.67	0.47	0.82
Educational level	0.29	1	0.59	Elementary	3	0.53	0.04	0.96
				Tertiary	18	0.73	0.55	0.86
Development level	19.25	1	0.00	Developed	6	0.93	0.86	0.96
				Developing	15	0.56	0.36	0.73
Student major	1.31	1	0.25	Diverse	5	0.60	0.28	0.85
				Medical	8	0.83	0.51	0.96

## 4. Discussion

Based on the above findings, we have discussed below the possible explanation of these outcomes (the combined and moderating effect of anxiety, depression, and stress prevalence), combining it with related prior literature. In addition, limitations are discussed for guiding follow-up studies in the future.

### 4.1. Prevalence of anxiety, depression, and stress

The present study is the first meta-analysis on the prevalence of anxiety, depression, and stress among remote learning students during the COVID-19 pandemic. It synthesized the evidence on the empirical studies associated with online learning and mental health problems, such as anxiety, depression, and stress during the COVID-19 pandemic period. After a rigorous literature search and screening, a total of 36 studies with 78,674 participants from 19 nations were included and generated 60 effect sizes (22 for anxiety, 17 for depression, and 21 for stress). This meta-analysis showed that the prevalence of anxiety, depression, and stress among remote learning students during the COVID-19 pandemic was high, 58, 50, and 71%, respectively.

Comparing the current result with previous analogous meta-analysis, one study found that the prevalence of anxiety, depression, and stress among remote learning students during the COVID-19 pandemic is significantly higher than that of the general population during the pandemic, students during the pandemic, and students before the pandemic. More specifically, Salari et al. (2020) found that the general population’s prevalence of anxiety, depression, and stress during the COVID-19 pandemic was 31.9, 33.7, and 29.6% *via* meta-analysis. In addition, Wang et al. (2021) conducted a meta-analysis study and concluded that the prevalence of anxiety, depression, and stress among general college students during the pandemic period was 29, 37, and 23%. As regards the meta-analysis before the outbreak of COVID-19, the prevalence of stress, anxiety, and depression among students was 32.9, 30.6, and 49.9%, respectively (Pacheco et al., 2017). All these meta-analyses indicate that many more remote learning students suffer from anxiety, depression, and stress than the general population and students during and before pandemic.

Based on the mentioned data comparison, student groups, online learning, and the COVID-19 pandemic are three factors that lead to a higher prevalence of anxiety, depression, and stress. First of all, children and young people nowadays are facing unprecedented challenges that are unacquainted with previous generations’ cognition, and it is exactly the time in one’s life that confronts the peak risk of suffering from mental health problems (Claveirole and Gaughan, 2011; Cunningham and Duffy, 2019). According to a study conducted by the American College Health Association (2014) based on 79,266 participants, students reported feeling anxious, depressed, hopeless, psychologically exhausted, and even considered suicide and self-harm. The stressors they are exposed to are not limited to academic pressure but social, financial, and lifestyle factors (Cunningham and Duffy, 2019). Therefore, the current result conforms to the previous finding that students are more vulnerable and prone to mental illness. Second, using technology may also be detrimental to mental health, though electronic devices have long been applied as everyday tools for people from all walks of life including students (Zeeni et al., 2018). Computer-related anxiety is not an up-to-date concept but was put forward decades ago, which refers to a non-rational anticipation of dread brought on by the notion of using the computer (Brosnan, 1998). It further derives from another concept, internet-related anxiety, including four aspects of specific anxiety caused by the internet: (1) internet terminology anxiety, (2) net search anxiety, (3) internet time delay anxiety, and (4) a general fear of Internet failure (Presno, 1998). Under the learning context, students who took online lessons reported anxiety, especially at the beginning of the courses (DeVaney, 2010). Apart from anxiety, stress is another mental health problem that resulted from the use of technology. Utilizing new information-communication-technologies (ICTs), such as laptops, mobile phones, and virtual education, can result in stress, which is referred to as “technostress” in modern parlance (Torales et al., 2022). Some researchers found that the most common technostress consequences are anxiety and depression (Chiappetta, 2017; Lin et al., 2017). Hence, it is understandable that online learning can trigger negative emotions, which is consistent with previous research (Zembylas, 2008). Last but not least, the COVID-19 pandemic is a non-negligible element that has exacerbated mental health problems, especially for students and young people (Meda et al., 2021; Kauhanen et al., 2022). A systematic review conducted by Meda et al. (2021) found that 87% of previous studies showed increased anxiety, depression, and mental distress among children and young people after the outbreak of the COVID-19 pandemic. Potential explanations for this phenomenon are numerous, including but not limited to remote learning (Almeida et al., 2021), increased social media use (Ellis et al., 2020), more stringent COVID-19 policies (Aknin et al., 2022), social isolation, worries about being infected, and restrained leisure activities (Meda et al., 2021). Thus, taking present and previous studies into consideration, student groups, online learning, and the COVID-19 pandemic are three factors that have given rise to the prevalence of anxiety, depression, and stress.

### 4.2. Moderating factors of anxiety, depression, and stress

In the moderator analysis, several variables are set as moderators in the current meta-analysis, including publication year, whether attending emergency online learning, educational level, development level, and students’ major. It is the first study to explore the moderating effect of study publication year on the prevalence of anxiety, depression, and stress among remote learning students during the COVID-19 pandemic. This meta-analysis concluded that the moderating effect of publication year was not significant, which also indicates that the prevalence of anxiety, depression, and stress scarcely variates with time after the outbreak of the COVID-19 pandemic. The result shows a different trend from that before the pandemic. Before the start of the COVID-19 pandemic, studies found that the level of anxiety and depression among adolescents increased substantially from 1997 to 2017 in China and from 2011 to 2018 in America (Twenge, 2020; Xin et al., 2020). The same result was also shown in the study focused on university students, which found that the rate of depression among American college students also increased from 2007 to 2017 (Lipson et al., 2019). The reason behind such difference may be hidden by the fact that the time duration after the pandemic is merely 3 years (from the end of 2019 until now), which does not have a prolonged chronological root as previous studies.

Whether attending emergency online learning is also an innovative variable studied in this meta-analysis. The result showed that the moderating effect of whether attending emergency online learning was significant for the prevalence of stress (the stress prevalence of students under the emergency online learning context was significantly higher than that of students under the traditional online learning context), but we failed to find the moderating effect in the prevalence of anxiety and depression among online learning students during the COVID-19 pandemic. Compared to traditional online education, the emergency pattern is usually a temporary shift to another delivery mode due to crisis circumstances (Ferri et al., 2020; Hodges et al., 2020; Fuchs, 2022). For this reason, emergency online teaching confronts various distinct challenges and obstacles from traditional online study, including restrained planning and preparation time, lack of faculty professional training, and unfamiliar access to a technological support system (O’Keefe et al., 2020). Thus, emergency remote education could exert a series of negative effects, such as anxiety, depression, distress, fear, and worry (El-Sakran et al., 2022). In the present study, the significant difference between traditional and emergency online learning in students’ perception of stress was identified on comparison with previous studies. However, the reason for showing no difference in online students’ perception of anxiety and depression may be aroused by the insufficient sample of related studies.

Educational level is the third moderator variable in this meta-analysis. The result showed that the moderating effect of the educational level was significant for the prevalence of anxiety and depression among e-learning students during the COVID-19 pandemic. The anxiety and depression prevalence of university students was significantly higher than that of the k-12 students. This result partly conforms to the finding in another meta-analysis, which found that people with a higher level of education experienced a higher level of anxiety, depression, and stress (Salari et al., 2020). There are two potential explanations. On the one hand, people with a higher educational level possess higher self-awareness in terms of their health (Zhang and Ma, 2020). On the other hand, the stressors confronting people with different educational levels are disparate. University students are exposed not only to academic pressure but more importantly to prolonged unemployment, along with financial insecurity and undermining self-esteem, which are all contributors to the increased rate of mental health problems such as anxiety and depression (Islam et al., 2020), which should not be taken into consideration for k-12 students. Nevertheless, no significant difference showed between tertiary and elementary students in the prevalence of stress during the COVID-19 pandemic online learning. A possible reason for the difference of the present study from the previous study is that the subjects of the present study are remote learning students while the subject in the other study is the general population (Salari et al., 2020).

As regards the development level, the result showed that the moderating effect of development level was significant for the prevalence of stress among e-learning students during the COVID-19 pandemic. The stress prevalence of students in a developed country was more than 1.5 times higher than their counterparts in a developing country. The current result is exactly opposite to the finding among the general population, which found that people in underdeveloped and developing countries suffer from more psychological problems (Salari et al., 2020). It is primarily because underdeveloped nations are associated with poorer treatment conditions, community inefficiency, and many other infectious diseases (Moghanibashi-Mansourieh, 2020; Salari et al., 2020; Sigdel et al., 2020). However, narrowing down the participants to students only, similar findings have been reported in China, which found the prevalence of sleep problems and suicide attempts among university students in eastern China (a relatively developed region) is higher than their counterparts in central and northeast of China (a relative developing region; Chen et al., 2022). A possible explanation may have originated from the actuality that students suffer from greater peer pressure in places with rich educational and economic resources due to better enrollment quality and more intense social competition (Liu et al., 2019; Chen et al., 2022). Hence, the prevalence of stress for distance learning students in developed countries was found to be higher than their counterparts in developing countries.

The last moderator variable in this meta-analysis is students’ major, which shows no significant difference between students in medical and other diverse majors in the prevalence rate of anxiety, depression, and stress during the COVID-19 online study period. This result is in discordance with the previous study. Reviewing preceding research comparing mental health between medical and non-medical students, there are two contrary findings before and after pandemic. Before the outbreak of the COVID-19 pandemic, more medical and non-medical students reported worse mental health status due to perceiving greater academic pressure (Al-Dabal et al., 2010). However, the result reversed after the spread of COVID-19 since medical students showed fewer mental health symptoms such as anxiety, depression, and stress than non-medical students, because they gained richer medical knowledge and had a higher sense of awareness (Xiong et al., 2021). Some scholars assumed that online study posed numerous challenges to medical education, since hands-on experience is of vital importance for medical students (Patra et al., 2021; Nikas et al., 2022), whereas the result of this meta-analysis did not indicate lacking hands-on experience leads to more mental problems among medical students. It is possibly because greater academic stress and richer medical professional knowledge cancel out, so there is no higher prevalence level of anxiety, depression, and stress among online medical students during the pandemic period.

### 4.3. Limitations and future study

Despite this study being a pioneering work exploring the prevalence of anxiety, depression, and stress among remote learning students during the COVID-19 pandemic, several limitations exist in the present meta-analysis. First, the number of studies on emergency remote learning is insufficient: only three for anxiety and stress and one for depression, which leads to the moderating effect of whether attending emergency remote learning on the prevalence of depression cannot be examined. Therefore, more searches under emergency remote learning can be collected to be further analyzed in the future. Second, there are only two categories for students’ major variables: medical and diverse. Students in different majors confront their own specific challenges that other disciplines cannot notice. Thus, scholars should also pay attention to students’ mental health in other majors, such as business, literature, art, and science. Third, only published articles were collected in this study. It is relatively difficult to avoid publication bias in meta-analysis since it is a secondary analysis based on abundant original literature. However, including unpublished articles could lower bias. Fourth, the current research is yet to cover all types of mental health problems. Only anxiety, depression, and stress were regarded as representative indicators reflecting remote learning students’ mental status; therefore, many psychological disorders, such as distress and self-injury, can catch attention in the future. Fifth, all original studies in the current meta-analysis were periodic, which could only reflect participants’ mental health over a period of time. However, psychological status changes as time goes on. Therefore, future follow-up studies could be conducted to explore the overall psychological impact of the COVID-19 pandemic on students in multiple disciplines over a more forward-looking period.

## 5. Conclusion

Over the past 3 years, the COVID-19 pandemic has triggered psychological problems afflicting people worldwide. This study conducted a meta-analysis based on 36 original articles and 78,674 participants across 19 nations and found that the prevalence of anxiety, depression, and stress among remote learning students during the COVID-19 pandemic was as high as 58, 50, and 71%, respectively. The prevalence rate of anxiety and depression among students in higher education was significantly higher than that of students in elementary education. Besides, significantly more medical students and students in emergency remote learning contexts suffer from mental stress than their non-medical and traditional distance learning counterparts.

Therefore, instructors, schools, and governments should take notice of the current situation and figure out ways to collaboratively help online learning students relieve anxiety, depression, and stress against the backdrop of the COVID-19 pandemic. Instructors, as the group with the closest connections to students, could alleviate students’ negative emotions by designing flexible coursework and test-taking, having organized online courses, and showing care about students’ mental health (Mohammed et al., 2021; Busch et al., 2022). From the schools’ perspective, professional programs for reducing students’ mental disorders, such as anxiety, depression, and stress, could be implemented. Besides, as a determining factor in students’ experience, the schools should shoulder the responsibility to provide school counseling services for faculty, students, and their family members (Karaman et al., 2021). In terms of governments, more macroscopic measurements can be implemented, including enhancing digital infrastructure and enriching e-content and e-resources, especially for students in less developed areas (Adnan and Anwar, 2020). Under the joint effort of instructors, schools, and governments, it is assumed that the symptoms of anxiety, depression, and stress can be relieved among remote learning students.

## Data availability statement

The raw data supporting the conclusions of this article will be made available by the authors, without undue reservation.

## Author contributions

TX is responsible for putting forward ideas, collecting and analyzing data, and drafting the paper. HW is responsible for supervising the whole process and revising the paper. All authors contributed to the article and approved the submitted version.

## Conflict of interest

The authors declare that the research was conducted in the absence of any commercial or financial relationships that could be construed as a potential conflict of interest.

## Publisher’s note

All claims expressed in this article are solely those of the authors and do not necessarily represent those of their affiliated organizations, or those of the publisher, the editors and the reviewers. Any product that may be evaluated in this article, or claim that may be made by its manufacturer, is not guaranteed or endorsed by the publisher.
